# On determining the mechanical nociceptive threshold in pigs: a reliability study

**DOI:** 10.3389/fpain.2023.1191786

**Published:** 2023-05-17

**Authors:** Felipe Rettore Andreis, Carsten Dahl Mørch, Winnie Jensen, Suzan Meijs

**Affiliations:** Center for Neuroplasticity and Pain (CNAP), Department of Health Science and Technology, Aalborg University, Aalborg, Denmark

**Keywords:** pressure algometry, pigs, pain, mechanical nociceptive threshold, reliability

## Abstract

**Background:**

A pressure algometer is a valuable tool for assessing the mechanical nociceptive threshold (MNT) in clinical pain studies. Recent research has turned to large animal models of pain because of the closer anatomy and physiology to humans. Although the reliability and usefulness of the MNT have been extensively validated in humans, similar data from large animals is still sparse.

**Objective:**

Therefore, the aim of the current study was to evaluate the reliability (within- and between-session) of MNT in the forelimb of pigs using a pressure algometer.

**Methods:**

Nine animals were used (23–40 kg), and MNTs were measured at both the right and left limbs at three different sessions, with three repetitions per session. The intraclass correlation coefficient (ICC) was used as a metric for relative reliability. The standard error of measurement (SEM) and coefficient of variation (CV) was used to assess absolute reliability. Systematic bias was also evaluated.

**Results:**

The average ICC was found to be 0.71 and 0.45 for the between-session and within-session, respectively. CV ranged from 17.9% to 20.5%, with a grand average of 19.1%. The grand average SEM was 249.5 kPa (16.6%). No systematic differences were found for the MNT between sessions, which suggests that there was no habituation to the stimulus.

**Conclusion:**

The reliability indices obtained in this study are comparable to results obtained in other species or anatomical regions and substantiate the use of the pressure algometer as a valuable tool to investigate the nociceptive system in pigs and translation to the human nociceptive withdrawal reflex.

## 1. Introduction

Pain is a multifaceted and subjective experience resulting from the intricate interplay between psychological, biological, and social elements (1). Because of its subjective nature, studies in humans rely on the subject's ability to express their pain experience through standardised questionnaires and quantitative scales. In animals, however, pain cannot be directly measured, and researchers can only infer the animal's pain state through surrogate behaviours (2).

Nociceptive threshold testing (NTT) is a well-validated method to investigate experimentally painful conditions in animals, such as allodynia (i.e., pain due to a stimulus that does not normally provoke pain), hyperalgesia (i.e., increased pain response to a painful stimulus), and to test the efficiency of analgesic compounds (3). NTT is stimulus-dependent and entails the application of a quantifiable stimulus to a particular body location until a behavioural or physiological response is noticed (e.g., withdrawal, vocalisation) (4).

There are mainly four types of stimuli used in NTT: mechanical, thermal, electrical, and chemical (5). Thermal and mechanical stimuli are the most adopted sensory modalities because they provide natural stimuli that are easy to control and can be applied on a continuous scale, while chemical stimuli need to be dosed and cause sustained stimulation (5). Mechanical stimulation can be further subdivided into static (triggered by pressure), dynamic (triggered by brushing), and punctate (triggered by touch) (6).

Most pain preclinical studies have been conducted in rodents (3) and therefore, numerous techniques have been developed to assess “pain-like” behaviour in this species [for a comprehensive review, please read (6)]. The almost sole dependence on rodents as preclinical models might be an important factor explaining the poor translational record of the pain field, and researchers suggested using larger animal models to bridge the translational gap between rodents and humans (7). Pigs, in particular, are promising models because they share many physiological and anatomical characteristics with humans (e.g., skin structure, sequence homology, metabolism, and nerve fibre classes) (8). The interest in pigs was highlighted in a recent systematic review that revealed a substantial increase in the number of studies looking at pain in pigs using various model types (i.e., evoked pain models, production procedures, naturally occurring pain and disease models) (9).

NTT testing has, over the last decade, been extended to larger animal species (e.g., calves, horses, sheep, and dogs) (10–14), and the reliability of these measures have varied significantly between different species and body sites (15–17). In pigs, prior studies have, however, focused on suitability and factors influencing mechanical nociceptive threshold (MNT). Giminiani et al. demonstrated the feasibility of using a pressure algometer for measuring MNT in pigs’ tails. Janczak et al. and Nalon et al. evaluated confounding factors using hand-held and limb-mounted algometers in assessing MNT in piglets and sows, respectively (16–18). To the best of our knowledge, no studies are currently available focusing on estimating the reliability of mechanical sensory testing in the pig. As the pig is gaining interest as a translational model, the aim of the present study was to quantify the reliability (within-session and between-session) of MNT using a pressure algometer in the forelimb of pigs.

## 2. Materials and methods

### 2.1. Animals

Nine adolescent female Danish Landrace pigs acquired from a commercial farm were included (23–40 kg). The animals were housed in pairs in iron enclosures with a 13:11 h light-dark cycle. Commercial food was provided twice daily, and nipple drinkers allowed the animals unlimited access to water. The room was maintained at ≈24°C. The study was approved by the Danish Veterinary and Food Administration under the Ministry of Environment and Food of Denmark (protocol number: 2020-15-0201-00514).

### 2.2. Habituation and training

The pigs were habituated and trained daily at roughly the same time (08:00 to 10:00 AM) to decrease stress levels and increase the method's reliability. The pigs were habituated to the stable, the caretakers, the researchers, the equipment and separation from the mate for one week after arrival at the facility. The pigs then underwent clicker training individually for one week to train them to stand still and accept the mounting of the boots. When both the active and dummy boot were mounted, the animal received a food bowl with their regular commercial food to allow them to relax and stand still. At the conclusion of the training period, no retraction of the limbs was observed during the mounting boot attachment to the leg. In the third week, measurements of the MNT were conducted while pigs were eating calmly by their food bowl with both boots mounted.

### 2.3. Instrumentation and procedure

**Figure 1:**
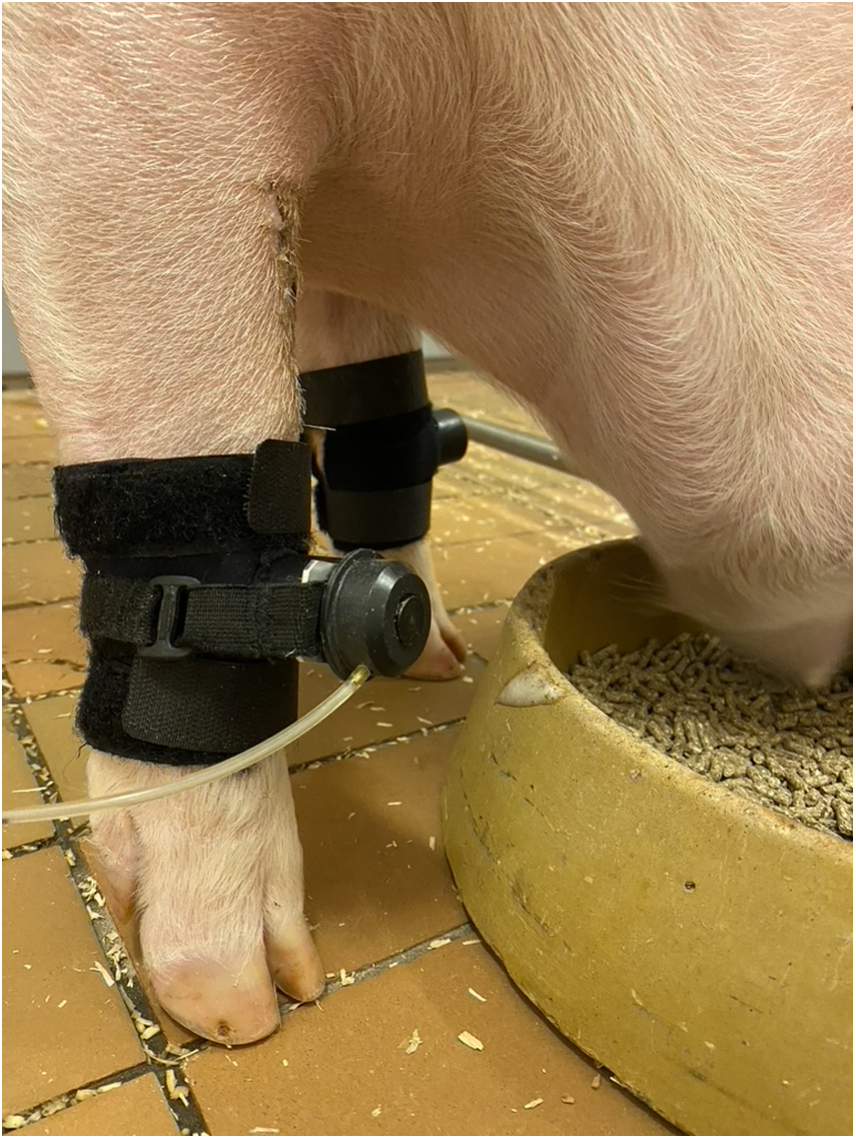
Mounting boots are attached to both limbs; one contains a dummy actuator and the other the active probe, which is fixated through a strap and buckle kit. The actuator contains a blunt-ended pin that works by pressing against the skin via air injection from a syringe.

The pressure algometer system consisted of an active actuator mounted in a placement boot on the animal's forelimb and connected to a strap and buckle kit to fixate the probe (ProdPro, Topcat Metrology Ltd, United Kingdom). A blunt-ended pin (2 mm diameter) protruded from the placement boot to allow the experimenter to apply pressure on the animal's limb. The blunt-ended pin was positioned approximately two cm below the middle carpal joint, roughly 45° from the sagittal plane. The pressure was manually induced via air injection from a syringe. The injection rate was controlled with the assistance of green and red lights that indicated if the pressure rate should be increased or decreased to maintain a constant slew rate of 2 N/s. The pressure algometer kit ensured a perpendicular angle between the pressure point and the skin surface. Finally, both limbs were tested, and while the mounting boot and actuator were placed on one limb, a dummy actuator and mounting boot were placed on the contralateral limb (see Figure 1).

**Figure 2:**
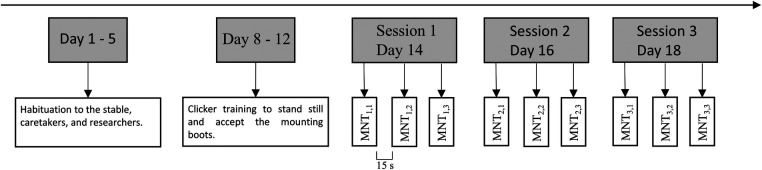
Experimental procedure for the reliability measurements. At each session, the animal is measured three times, at both limbs.

The pressure was increased gradually until a foot lift was visually observed at which the respective force level was annotated. Stimulation was also immediately stopped when the cut off force of 25 N was reached. Three measurements were obtained on each limb at each experimental session, with a minimum rest interval of approximately 15 s. Finally, the animals were measured for three days, with a one-day interval between each measurement day. In total, 162 measurements were obtained, representing nine animals measured three times per session, on three sessions, at both limbs. The experimental procedure is described in Figure 2.

### 2.4. Statistical analysis

The force measurements were converted to pressure by dividing the force by the probe area. The statistical analysis was split into two parts: the first to assess internal consistency (within-session) reliability and the second to assess stability (between-session) reliability (19). Within and between-session systematic errors were tested with the one-way repeated measures ANOVA.

The relative reliability was determined by calculating the intraclass correlation coefficient 2-way mixed-effects model type absolute agreement (ICC_2,*k*_) where *k* indicates the average of three repetitions. The average form, rather than the single measurement of ICC, was selected because a few repetitions are often performed in MNT. ICC values were interpreted based on a previously proposed category, according to which an ICC between than 0.81 and 1.00 is considered almost perfect, from 0.61 to 0.80 it is considered substantial, values between 0.41 and 0.60 are considered moderate, and below 0.40 it is considered unacceptable (20).

The absolute reliability was evaluated by the coefficient variation (CV) and standard error of measurements (SEM). CV was computed by the within-subject standard deviation as a proportion of the within-subject mean, indicating the stability of a measure across repeated trials (21). The SEM was defined as $SEM=SD\sqrt{1-ICC}$ and reflects the precision of individual scores on a test, meaning that it can be considered an estimation of expected random variation when no real change has occurred.

Finally, results are presented as mean and SD unless otherwise specified. The adopted significance level was 0.05, and the assumptions of normality and homoscedasticity were verified through residual analysis (Q–Q plots and histograms). Statistical analysis was performed with R software (22).

## 3. Results

There were no significant differences between the average MNTs of the left and right limbs (session 1: *p *= 0.95, session 2: *p* = 0.54, session 3: *p* = 0.60); therefore, the following analysis was performed on a pooled dataset for the left and right limb.

**Figure 3:**
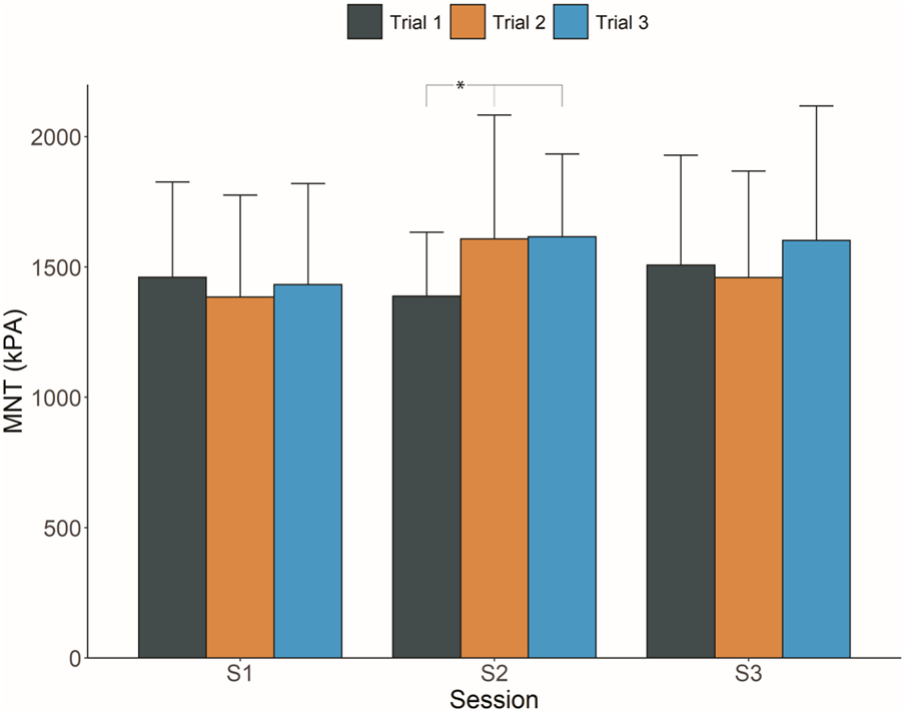
Mean mechanical nociceptive thresholds (MNTs) across every trial and session. The error bars represent standard deviations. *p < 0.05.

The average MNT for all sessions and trials are shown in Figure 3. The MNT was not significantly different between trials in sessions 1 and 3; however, there was a significant difference in MNT between trials in session 2 (*p* = 0.02). The post-hoc analysis revealed a lower MNT in trial 1 than in trials 2 and 3. There were no significant differences in average MNT for the between-session analysis (i.e., session 1 vs. session 2, session 2 vs. session 3, and session 1 vs. session 3).

The results from the MNT reliability analysis for the within- and between-session reliability analysis are shown in Table 1. Six relative reliability measures were obtained with ICC values ranging from 0.30 to 0.81. The average ICC for the within-session analysis was 0.71, while the average ICC for the between-session analysis was 0.45. Interestingly, all values of ICC from the between-session analysis are lower than the ones from the within-session analysis, demonstrating a higher day-to-day variability compared to the variability within the same day.

**Table 1:** Intrarater, within-session and between-session reliability of mechanical nociceptive threshold.

	Within-session analysis			Between-session analysis		
	Session 1	Session 2	Session 3	Sessions 1–2	Sessions 1–3	Sessions 2–3
Mean ± SD	1426 ± 376	1537 ± 368	1522 ± 447	1481 ± 374	1474 ± 414	1530 ± 407
RM-ANOVA	*p *= 0.73	*p* = 0.02*	*p* = 0.41	*p *= 0.06	*p *= 0.13	*p *= 0.82
CV_%_	18.1	17.9	19.8	18.9	19.9	20.5
ICC_2,*k*_ [95% CI]	0.68 [0.40–0.85]	0.66 [0.37–0.84]	0.81 [0.64–0.91]	0.55 [0.30–0.71]	0.51 [0.23–0.69]	0.30 [0–0.56]
SEM [absolute vs. %]	215.8 [15.1%]	201.8 [13.1]	195.6 [12.9]	249.4 [16.8]	284.9 [19.6]	340.8 [22.3]

*Indicates *p* < 0.05.

The obtained measures of absolute reliability were CV and SEM. The CV, expressed as a percentage, ranged from 17.9% to 20.5%. The average within-session CV was 18.6%, while the mean between-session CV was 19.7%. The SEM ranged from 195.6 kPA (12.9%) to 340.8 kPA (22.3%), with an average of 204 kPA (13.7%) for the within-session and 291.7 kPA (19.5%) for the between-session reliability. The measures of absolute reliability indicate a lower reliability of the data from day to day compared to the variability of multiple repetitions within the same day.

Finally, the overall reliability between the legs was computed, resulting in an ICC of 0.61 [95% CI: 0.43–0.73].

## 4. Discussion

The assessment of pain behaviour in pigs has been mostly concerned with pig production procedures such as tail amputation (23) and castration (24). Biomedical research has also seen an increased interest in pigs as subjects for translational pain models (25, 26), where the assessment often relies on evoked responses of mechanical and thermal nociceptive stimulation. In order for a behavioural model for nociception to be useful, the measurement must meet five different requirements: specificity, sensitivity, validity, reliability, and reproducibility (5). The literature on the reliability of quantitative-sensory testing in these animal models is still sparse. Therefore, this study focused on estimating the reliability of MNT longitudinally in the forelimb of pigs.

### 4.1. Relative reliability

Six relative reliability measures were obtained with one almost perfect ICC (>0.81), two substantial ICCs (0.61–0.80), two moderate ICCs (0.41–0.60), and one unacceptable ICC (<0.40). The average ICC for the within-session analysis was substantial (i.e., 0.71), while the average ICC for the between-session analysis was moderate (i.e., 0.45), confirming that a pressure algometer is a valuable tool in assessing the nociception in the forelimb of pigs. We have also found that the within-session reliability was higher than the between-session reliability, indicating that the most considerable variability occurs between different days, even though measurements were obtained at roughly the same time every day to control for circadian patterns. This result could be explained by subtle changes in the position of the blunt-ended pin. Contrary to the hand-held algometer, where the exact position can be seen, the placement boot masks a clear view of the pin location.

Earlier studies were carried out to quantify the reliability of these methods in other species or at different body locations of the pig, such as the tail (16), where the authors used a hand-held pressure algometer in three distinct tail regions for animals with different ages and obtained on average, ICCs ranging from 0.33 to 0.46, depending on the tail region. A study investigating mechanical thresholds at the back of the metacarpus in piglets’ legs found across different days ICC values in the range of 0.29 to 0.65 (18). The results obtained in this study indicate that in terms of relative reliability, our method is consistent with previous reports and substantiates the use of MNT in large animals. It must be stated that human studies tend to find higher reliability coefficients; for instance, in a study investigating interrater reliability of pressure pain threshold, the authors found high ICCs (>0.92) for several body sites such as wrist, leg, neck and back (27). It is expected that human studies display higher reliability since instructions can be given such that the participants specifically respond to a painful stimulus. In animals, however, one cannot be sure whether the animal is responding to a noxious sensation or other sensations. Another likely reason for higher ICCs in humans is that humans offer a more heterogeneous sample and therefore larger individual differences. As relative reliability is dependent on the between-subjects variability, this, in turn, can result in a higher ICC.

### 4.2. Absolute reliability

Regarding absolute reliability, we obtained a grand-average CV of 19.1%, lower than previous studies that found an MNT CV of 25.5% (17) and 35% (14) for the limbs of pigs and dogs, respectively. A CV of 19.1% can be considered low and is comparable to values obtained in human studies (14.6% for the leg and 17.7% for the arm) (28). Only a few studies reported the SEM, making the comparison with existing literature challenging. Still, the SEM is an important parameter to be compared with future studies as it indicates the precision of individual scores on the test (29). The grand-average SEM of 249.5 kPA obtained in this study is considerably higher than the 93 kPA obtained in the leg of humans (27), which can be explained by the fact that SEM tends to increase at higher scale values (20), and pigs have a higher MNT than humans. Therefore, to allow for comparison across different species, we also calculated the SEM in terms of percentages of the mean, which resulted in an average value of 13.7% and 19.5% for the within- and between-session, respectively. These values are comparable to human studies (20).

No systematic differences in MNT were observed between the sessions, suggesting no effect of habituation (i.e., increased thresholds) or sensitisation (i.e., decreased thresholds). A systematic difference was observed only within session 2, where MNT increased in trials 2 and 3, suggesting some adaptation has occurred. Still, the lack of systematic changes between sessions highlights the importance of adequate training of the animals for the task prior to the experiment so that measurements are not obtained in a period where the animal's familiarisation curve is changing. A prior study investigating MNT in dairy cows reported that pre-test habituation decreased the variability and increased the reliability of MNT (30). In sows, a study assessing anatomical and methodological factors influencing MNT reported increased MNTs over measurement days up to a stabilisation in the fourth and fifth day, indicating habituation to the stimulus (17). We did not observe a difference in the average MNT between the right and left limbs; however, contradictory evidence exists in the literature, with studies also reporting no left-to-right differences in MNT (31) and others reporting different values on the left vs. right side of the body, which might be a result of left- or right-side dominance (32).

### 4.3. Methodological considerations

An advantage of the “remotely-controlled” actuator used in this study is that the animal has no visual cue when the stimulus is given, which can generate anticipation of the stimulus (17). Another factor concerning the device that may influence the measurements’ reliability is the pressure application rate; the device uses a light system (green and red diodes) to indicate if the pressure is at the selected level. We observed that prior training with the device was enough for the researcher to keep the pressure rate stable during the experiment. Still, further improvements could include computer-controlled algometers with fixed pressure rates.

The limbs are particularly important in neuropathic pain models, where the disease is induced by some form of peripheral nerve injury (33). In animals, it is challenging to make the subjects stay still long enough to obtain accurate measures, especially in large animals. For instance, rodents can be immobilised with the hand to obtain the measurements (34). A “forced” immobilisation in large animals would be practically impossible and can generate stress-induced analgesia, affecting the measurements by increasing thresholds (35). Still, recent studies have shown the feasibility of assessing the nociceptive system in the limbs of pigs using von Frey filaments (36), laser stimulation (37), and mechanical stimulation (38). The latter used a perforated test platform to which the animals were acclimatised. In our study, the animals were tested in their pen, and a food bowl was sufficient to keep them standing still for the duration of the task.

It must be noted that several factors can influence the MNT; therefore, caution must be taken when translating the results from this experiment to other studies. Previous studies demonstrated that mechanical threshold increases with larger tip diameters (39) and time of the day, where thresholds are higher in the morning than in the afternoon (17). The range of MNT values obtained in this study is similar to other studies in pigs at the same weight range (38), but it is far smaller than MNTs in the limbs of heavier animals. In pigs weighing an average of 267 kg, thresholds of 16,500 kPA were observed (31). Therefore, the direct comparison of MNT between studies should also consider the effect of animal weight, as mechanical thresholds are positively correlated with body weight (18).

This study was conducted only on female pigs for two reasons. First, female subjects are underrepresented in preclinical pain research (40), despite the fact that the majority of chronic pain sufferers are female (41). This trend resulted in a male-based literature (42). The second reason relates to swine housing. Due to the fact that the animals in this experiment were housed in pairs, it is known that entire male pigs tend to exhibit more aggressive behaviour and fighting activity, particularly during puberty (43), which could impede the continuation of the study. Additionally, housing mixed-sex groups also result in more aggressive behaviour than housing only females (44). Sex differences in MNT have been investigated in dogs (39) and piglets (18), and none of the studies reported significant differences between males and females.

The effect of different examiners on reliability was not investigated in this study and should be considered in future experiments, as different examiners can display significantly different reliability levels that may be related to the examiner's experience or timing in detecting avoidance reactions (15).

## 5. Conclusion

The aim of the present work was to quantify the reliability (within-session and between-session) of mechanical nociceptive threshold (MNT) using a pressure algometer. This study indicates that mechanical nociceptive testing through a pressure algometer is a reliable research tool for investigating nociceptive thresholds in the limbs of pigs. Measures of absolute and relative reliability were superior to other animal studies and comparable to reliability studies performed in humans. Lastly, the absence of systematic differences between sessions corroborates the need for proper training of the animals prior to obtaining measurements.

## Data Availability

The raw data supporting the conclusions of this article will be made available by the authors, without undue reservation.
